# Acute internal medicine as the engine room of the modern hospital: The Midland Metropolitan University Hospital blueprint

**DOI:** 10.1016/j.fhj.2026.100534

**Published:** 2026-06-25

**Authors:** Sarbjit Clare, Jane Ho, Rachel Barlow

**Affiliations:** a Sandwell and West Birmingham NHS Trust, United Kingdom; b HKS Architects, London, United Kingdom

**Keywords:** Acute Internal Medicine, Hospital design, Midland Metropolitan University Hospital, New Hospital Programme, Emergency operational flow

## Abstract

Modern hospitals are increasingly defined by their ability to manage undifferentiated, complex acute medical demand rather than elective programmes or specialist services. The Midland Metropolitan University Hospital (MMUH) opened in October 2024, and is one of the first major UK acute hospitals deliberately designed around an acute internal medicine (AIM)-centred model. This article describes the clinically led design principles underpinning MMUH, how AIM was positioned as the operational core of the hospital, and reports early results demonstrating improvements in flow, bed occupancy, emergency access, workforce resilience, mortality and infection control. Despite consolidating two legacy hospital sites into a single building and opening with 100 fewer acute beds, MMUH has delivered measurable operational improvements within months of opening. These figures provide a compelling blueprint for the New Hospital Programme (NHP), demonstrating how embedding AIM at the architectural and strategic heart of hospital design can enhance safety, efficiency and patient outcomes. These findings represent early outcomes, and longer-term follow-up is required to assess the durability, sustainability and generalisability of the observed improvements.

## Introduction

Debates about ʻmodern hospitals’ often highlight surgical technology, diagnostics, or elective recovery. Yet the true test of a contemporary acute hospital lies in the management of the undifferentiated, acutely unwell patient. Emergency demand is increasingly characterised by older, frailer patients with multimorbidity, chronic disease exacerbations, delirium, falls and sepsis. These presentations rarely align with single organ specialties at the point of arrival.

Acute internal medicine (AIM) is uniquely positioned to respond to this demand as it provides senior physician assessment, rapid diagnostics, stabilisation, risk stratification and decisive clinical leadership for the majority of emergency medical admissions. As the ʻacute medical take’ increasingly defines hospital workload, AIM has become the operational engine room of the acute sector. When appropriately resourced and embedded into hospital design, AIM improves flow, reduces avoidable admissions, protects elective pathways and enhances safety. When under-resourced or housed in poorly designed environments, the consequences are evident across the NHS: overcrowded emergency departments, exit block, prolonged length of stay, cancelled operations, long waits, staff burnout and compromised safety.

Recognising this, the Midland Metropolitan University Hospital (MMUH), one of the largest new acute hospitals to open in England in more than a decade, was deliberately designed around an AIM-centred model.

Rather than retrofitting acute services into a traditional hospital layout, AIM informed the architectural design, clinical pathways, diagnostic adjacencies, workforce model and operational strategy from the outset. This article describes the MMUH model and presents early operational and patient outcomes demonstrating the impact of an acute-first, AIM-led approach.

### The Midland Metropolitan University Hospital model – the journey to a new acute hospital

**Fig. 1:**
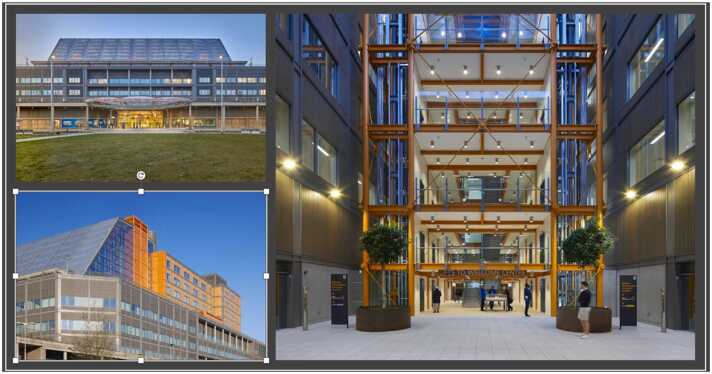
Midland Metropolitan University Hospital (MMUH).

MMUH forms part of Sandwell and West Birmingham NHS Trust (SWBH), serving one of the most diverse and socioeconomically deprived populations in England, and functions within a well-established integrated care system. It consolidates acute and emergency services previously delivered at City Hospital and Sandwell General Hospital into a single purpose-built site^1^ (Fig. 1).

Photographs reproduced with permission from Paul Raftery as per SWBH Licence © Paul Raftery.

The project encountered numerous national challenges: the collapse of construction contractor Carillion, the UK’s departure from the European Union, the COVID-19 pandemic, change to legislation due to the Grenfell Tower fire, supply chain shocks associated with the war in Ukraine, and unprecedented inflation affecting construction and energy costs. Despite these pressures, MMUH opened in October 2024 with 736 acute beds, including a deliberate reduction of 100 acute beds compared with the combined legacy sites. The move required meticulous planning, clinically led assurance and pathway redesign to ensure safety and operational continuity.

### AIM at the Helm – clinical and architectural partnership

A major determinant of MMUH’s early success was the deep and sustained involvement of clinicians, particularly acute physicians, throughout the design process. Working closely with HKS Architects, Cagni Williams Associates and Sonnemann Toon Architects, the clinical team shaped the building around real-world clinical logic rather than adapting to a traditional architectural template.^2^, ^3^

The core design question was not: ʻWhat should the building look like?’ but rather: ʻ*What do acutely unwell patients need, how will they move, and what do clinicians need to care for them effectively?*’

As a result, MMUH incorporates:

• A unified acute floor housing the emergency department (ED), acute medical unit (AMU), same-day emergency care (SDEC), acute surgery and cardiology
• Co-located diagnostics including imaging, point-of-care ultrasound (POCUS) and point-of-care testing (POCT)
• Vertically stacked floorplates connecting acute care, critical care and theatres
• Modern inpatient wards with 50% single rooms
• Clear wayfinding and separate circulation routes for patients, staff and visitors

A dedicated MMUH programme company, including AIM leadership, oversaw delivery and transition, allowing clinical and operational teams to stay focused on core pressures such as winter demand and industrial action.

Prior to opening, 41 acute clinical pathways were tested through rigorous tabletop exercises, specific on-site simulations, time and motion studies, emergency response drills and ʻday in the life’ scenarios to ensure that the estate supported safe care.

On the day of patient transfer, an AIM‐led patient census identified that only 327 of 736 inpatients required transfer, just 44% from the legacy sites. This validated the principle that early senior decision making and confident risk management reduce avoidable inpatient occupancy, highlighting the need for clinicians skilled in assessing uncertainty and determining who truly requires an acute bed. The census methodology has since attracted national interest.

## The MMUH acute model

### A unified acute floor (Fig. 2)

The acute floor brings together:

• Adult and paediatric EDs
• AMU with 108 beds
• Medical SDEC, including frailty
• AIM-led 32-bed short stay unit
• Acute surgery and cardiology
• Co-located diagnostics
• A dedicated emergency medical resuscitation team (EMRT)

**Fig. 2:**
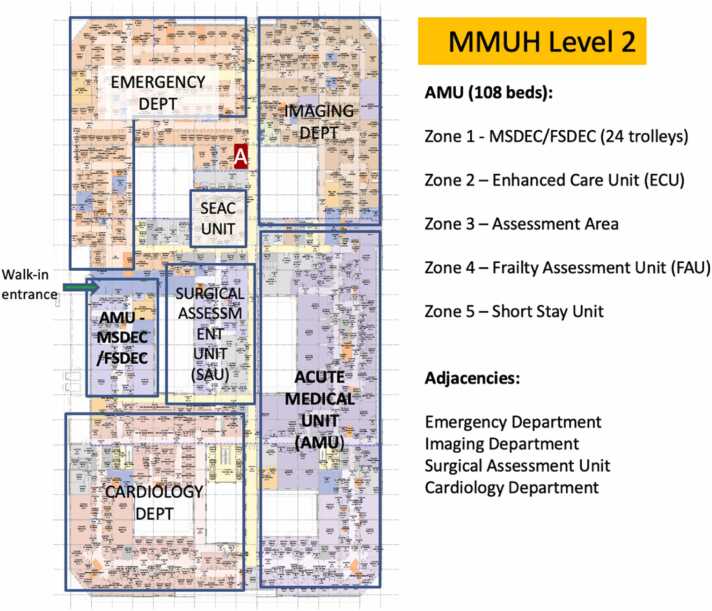
MMUH Level 2 – the acute floor.

Reproduced with permission from SWBH NHS Trust.

AIM leads the largest section of this floor, comprising an enhanced care unit, assessment zone, frailty assessment, short stay unit and medical SDEC. Theatres and critical care are situated directly above for rapid escalation. Separate circulation routes for staff, patients and visitors enhance efficiency and safety, as well as privacy and dignity.

### Diagnostics at the point of need

Imaging, laboratory services and POCT are integrated within the acute floor. AIM clinicians deliver rapid POCUS assessment, expediting diagnosis, supporting early decision making and diagnoses, and reducing avoidable admissions.

### Expanded SDEC and short stay capacity

The medical SDEC footprint includes 30 chairs, three consulting rooms, and flexible space for 24–36 trollies. The unit accommodates higher-acuity patients, ensuring timely senior review, investigations and treatment.

A separate surgical SDEC and surgical assessment unit (SAU) are housed on the acute floor.

The AIM-led short stay unit focuses on patients with an expected LOS under 72 h, supported by multiple daily consultant reviews.

### Workforce consolidation and resilience

Centralising acute services enabled a strengthened workforce model. Investment into AIM and general internal medicine (GIM) was critical to delivering 7‐day robust senior care.

• AIM consultant establishment increased from 12 to 18 whole time equivalents
• Robust 7‐day senior coverage for both front and back door
• Expansion of GIM teams to support a generalist approach
• AIM consultants now hold the resident medical officer bleep, ensuring immediate senior support

Prior to the move from the legacy estates, consultant cover on the AMU combined AIM and GIM responsibilities out of hours, and comprehensive 7-day consultant cover across SDEC and the medical wards was not established. Workforce consolidation and investment at MMUH have since enabled daily acute medicine leadership on the AMU and robust 7-day consultant cover for both SDEC and all medical wards.

Residents redesigned their rotas into 6-week AIM blocks, improving continuity, training and patient experience. Weekend senior and resident presence now mirrors weekday support.

### Modern ward infrastructure

**Fig. 3:**
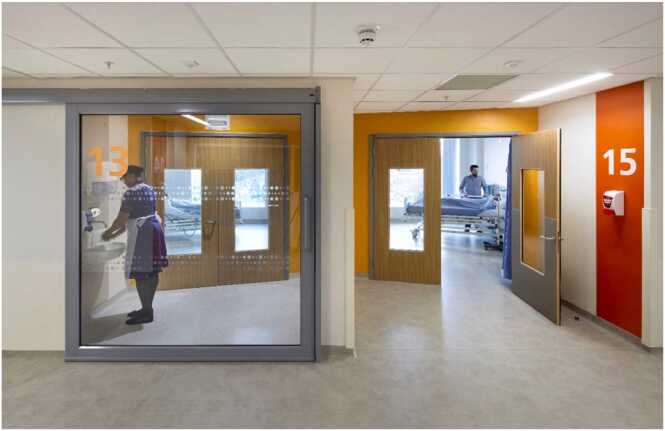
Photograph of single side rooms including a negative pressure side room.

With 50% single rooms, en-suite facilities, infection prevention-conscious layout, strong natural light and efficient wayfinding, the ward environment supports safety and dignity. An increase in the number of positive and negative pressure rooms was intentionally created (Fig. 3).

Photographs reproduced with permission from Paul Raftery as per SWBH Licence © Paul Raftery.

This contrasts with the antiquated legacy estate; City Hospital built in 1889 had limited isolation capacity and was severely impacted during the COVID-19 pandemic.

### Staff-centred environments

Enhanced rest spaces, optimal sightlines and more efficient operational flow have contributed to improvements in staff morale, retention, improved multidisciplinary working and perceived workload.

## Results

Despite opening immediately before winter, early internal and external evaluations demonstrate consistent positive trends.

### Improved emergency access performance

**Fig. 4:**
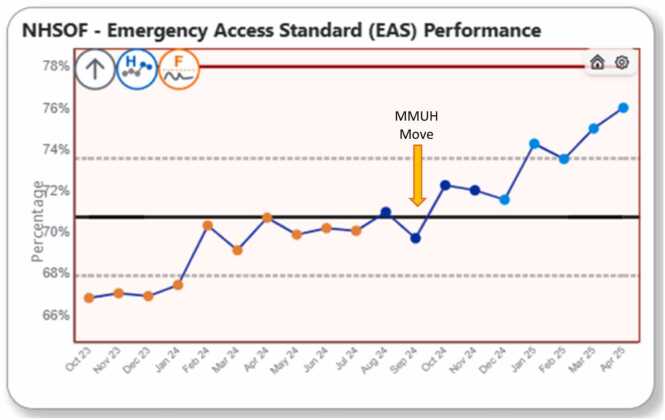
NHS Outcomes Framework (NHSOF) Emergency (EAS) performance.

Reproduced with permission from SWBH NHS Trust. Month on month since MMUH opened, EAS performance has shown a consistent improvement.

• Emergency Access Standard performance improved by **10–13%** compared with legacy sites (Fig. 4), reflecting improved flow from ED to AMU and inpatient wards.

### Reduction in bed occupancy

**Fig. 5:**
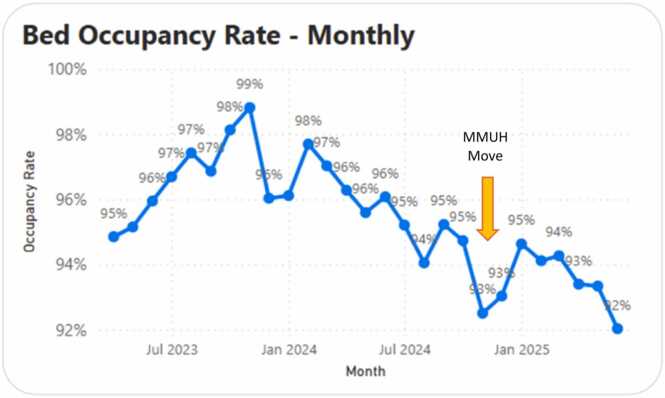
Bed occupancy rate – MMUH move October 2024.

Reproduced with permission from SWBH NHS Trust.

• Bed occupancy decreased from **∼99%** (legacy sites) to **∼92%** post‐move (Fig. 5). This reduction enables sustained ED outflow without the need for opening escalation areas. A reduction of decision to admit (DTAs) from approximately 50 pre-move to 0–15 post-move was realised.

### Reduction in non-elective length of stay (LOS)

**Fig. 6:**
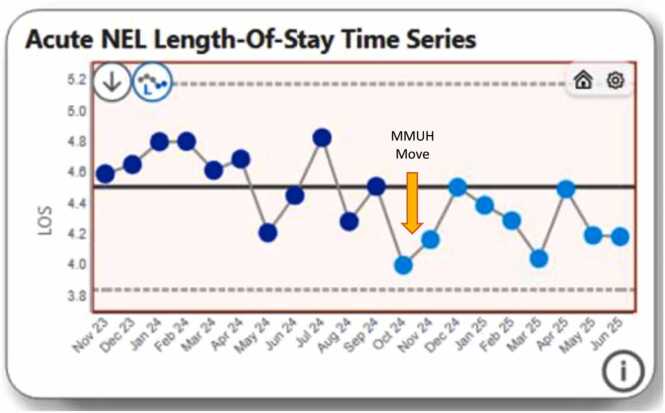
Acute non-elective length of stay.

Reproduced with permission from SWBH NHS Trust.

• Non-elective LOS has shown a sustained downward trend since consolidation (Fig. 6) with a reduction of approximately **1.5 days** for medical patients over 65 years.

### SDEC admission conversion rates reflect appropriate case mix

• Pre-move admission conversion: **3–5%** and post-move conversion: **∼13%,** reflecting appropriate streaming of higher‐acuity patients while avoiding unnecessary admissions.

### Medical short stay utilisation

**Fig. 7:**
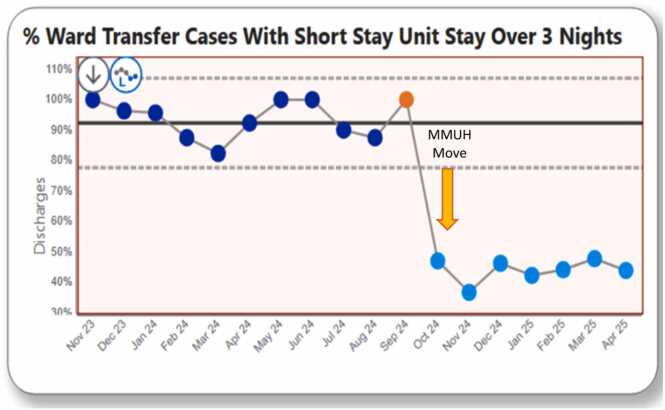
% Ward transfer cases with short stay unit over 3 nights.

Reproduced with permission from SWBH NHS Trust.

• Improved throughput and correct cohorting reduced inappropriate downstream short stay patient transfers and enabled the closure of 16 GIM beds during summer 2025 (Fig. 7).

### Improved clinical quality data markers, reduced acute take, improved clinical support and leadership

• ‘Acute medical take’ reduced from **∼90** pre-move to **60–70** patients per 24 h post-move, attributed to AIM consultants holding the bleep. This has subsequently led to:
  ￮ Enhanced and appropriate SDEC streaming
  ￮ Senior-led risk stratification
  ￮ Improved short stay utilisation
  ￮ Increased utilisation of community services including virtual wards and hospital at home services, avoiding hospital admission
  ￮ Improved relations between AIM, ED and community services

The MMUH Society Acute Medicine Benchmarking Audit (SAMBA) 2025, which measures quality indicators in all acute medical units within the UK, has demonstrated significant improvement from previous years in all three quality iIndicators since the move to MMUH.

Quality indicator 1, which measures observations within 30 minutes, has increased from 89% to 94.2% (median 83.3%), quality indicator 2 – time to see a clinical decision maker – has increased from 52.5% to 90.8% (median 79.4%) and the third quality indicator, which measures time to see a consultant in line with Society for Acute Medicine (SAM) and Royal College of Physicians (RCP), guidance has increased from 39% to 66% (median 53.2%).

### Workforce stability and improved rotas

**Fig. 8:**
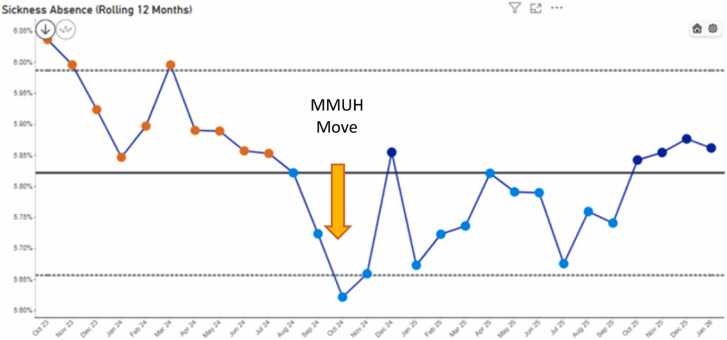
Sickness absence.

Reproduced with permission from SWBH NHS Trust.

• Resident doctor feedback shows improved training, continuity and morale under the 6-week acute block rota. Resident feedback was presented at the 2025 RCP Med+conference, and the team was awarded best presentation in health services and sustainability policy and workforce development in recognition of the grass roots movement. Weekend discharge rates now mirror weekday activity due to the robust 7-day consultant and resident cover.
• Teams such as ITU, maternity, ED and AIM are now under one roof, enabling teamwork, collaboration and robust rotas. One ED consultant’s feedback was: ʻWe are all together now in an amazing building which makes a huge difference to how we work and behave. Level 5 is perfect to get away from the intensity of the ED and get some downtime.’
• Organisational sickness absence has reduced since the move (Fig. 8). This improvement is attributed to the new environment, including single side rooms that support infection prevention, access to open, light-filled staff spaces with retail amenities, and dedicated rest areas on Level 5, alongside a strengthened organisational focus on staff wellbeing.

### Infection control resilience

• No medical beds were closed due to infection outbreaks during the first winter and staff sickness did not peak to typical winter levels.

### Patient safety and mortality

**Fig. 9:**
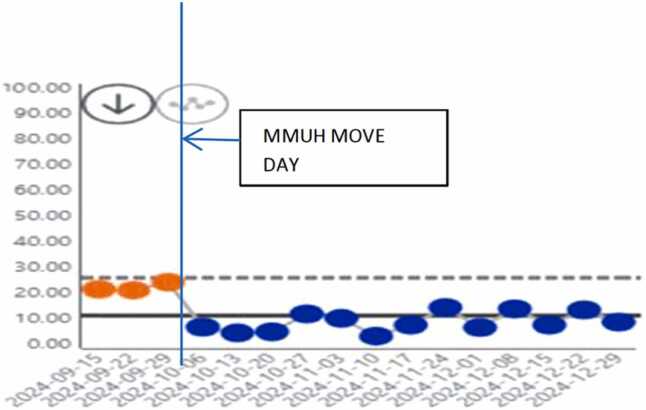
Deaths requiring a SJR pre and postmove.

Reproduced with permission from SWBH NHS Trust.

• Patients’ feedback indicates a coherent acute care pathway. One sickle patient stated ʻfor me the pathway straight to the SCAT centre, avoiding the ED and the long waits is the best part. Getting to see my specialist is critical for me.’
• Post-move, a reduction in the Summary Hospital-level Mortality Indicator (SHMI) from 110 to 95 was observed, as well as a reduction in the number of medical examiners concerns requiring a full Structured Judgement Review (SJR) (Fig. 9).

## Discussion

MMUH provides early evidence that designing a hospital around AIM principles can transform operational performance, workforce resilience, patient flow and safety. A unified acute floor with co-located diagnostics, vertically stacked critical care adjacency, and purpose-built short stay and SDEC capacity establishes an efficient acute ecosystem. These features reduce delays, shorten diagnostic pathways and accelerate senior-led decision making.

A critical lesson is the value of clinical leadership throughout design. When acute physicians, clinical teams and architects partner, they can shape the estate; the building reflects clinical logic rather than architectural convention. This contrasts with many Victorian and mid-20th-century hospitals, where dispersed services force staff to work around the estate, rather than the estate supporting staff. The MMUH design zoning by acuity, embedding diagnostics, reducing travel distances and enabling rapid escalation, demonstrates what is achievable when clinicians drive the process.

A dedicated MMUH programme team, including AIM, was central to its success, enabling timely delivery by protecting the work from daily operational pressures.

The findings also reinforce the value of a generalist model. Only 20% of patients require ‘single organ’ needs, suggesting that broad-based generalism is essential for the sustainability of the future of medicine.^4^ Where so many organisations underutilise GIM dually trained medical staff, SWBH proactively invested and promoted GIM as a key specialty for the acute hospital.

There are limitations. MMUH is still in its early operational phase, and some improvements may reflect ʻnew hospital uplift’. Further evaluation over multiple years is required, particularly through successive winters. Cultural integration following consolidation of two sites will also take time. However, the scale and speed of improvement suggest that the benefits arise primarily from deliberate structural and operational design.

The move to MMUH has already delivered measurable improvements in the quality of care. As services continue to embed and fully utilise the potential of this purpose-built environment and modern equipment, there is a significant opportunity to move from improvement to excellence, and ultimately to deliver exceptional care. At times within the NHS, we assume that certain changes are not possible; however, this new environment offers an opportunity to challenge those assumptions and rethink what can be achieved. The opportunity now is not simply to do the same work in a new building, but to rethink what is possible and set a new standard for care.

Beyond clinical performance, MMUH positions itself as *#MoreThanAHospital*, as a beacon of hope, tackling health inequalities, and acting as a catalyst for local regeneration. The project has already created over 200 local jobs, has a new learning campus and will deliver affordable housing, improved transport links, and new opportunities for local businesses for a community that urgently needs it.^5^

For the New Hospital Programme (NHP), the implications are clear. New hospitals must not replicate outdated layouts with fragmented acute services and poor diagnostic adjacencies. An AIM-centred blueprint, unified acute floors, senior‐led streaming, expanded SDEC/short stay capacity and embedded diagnostics should form the minimum standard for acute hospital design nationally. The MMUH experience demonstrates that when the acute engine room is deliberate, central and clinically led, the whole system benefits.

## Conclusion

MMUH demonstrates that an acute hospital designed around the principles of AIM, clinically led, operationally coherent and focused on the undifferentiated patient, can deliver measurable improvements in flow, safety and efficiency within months of opening.

As the NHS embarks on the largest hospital-building programme in a generation, the MMUH blueprint offers a compelling case for embedding AIM at the heart of every new acute hospital. When the acute ʻengine room’ works, the whole system works.

## Key points

• Acute internal medicine should be the central organising principle for new acute hospital design.
• Unified acute floors with co-located diagnostics improve flow and safety.
• Large SDEC and short stay capacity reduce unnecessary admissions and length of stay.
• Senior decision makers present 7 days per week improve outcomes and reduce the acute take.
• Modern ward infrastructure and single rooms support infection control and patient dignity.
• MMUH demonstrates improved emergency access, lower occupancy, reduced length of stay, improved patient safety and greater workforce stability.
• The New Hospital Programme should adopt AIM-led design standards nationally.

## CRediT authorship contribution statement

**Sarbjit Clare:** Writing – original draft. **Jane Ho:** Writing – review & editing. **Rachel Barlow:** Writing – review & editing.

## Funding

This work/viewpoints and research did not receive any specific grant from funding agencies in the public, commercial, or not-for-profit sectors.

## Declaration of Competing Interest

The authors declare that they have no known competing financial interests or personal relationships that could have appeared to influence the work reported in this paper.
